# A Case of Mediastinitis Due to Fulminant Group A Streptococcal Infection

**DOI:** 10.70352/scrj.cr.25-0440

**Published:** 2026-03-31

**Authors:** Yuta Abe, Naoki Haratake, Takahiro Sato, Miyuki Abe, Yohei Takumi, Kenji Sugio, Atsushi Osoegawa

**Affiliations:** Department of Thoracic and Breast Surgery, Oita University Faculty of Medicine, Yufu, Oita, Japan

**Keywords:** mediastinitis, group A *Streptococcus*, streptococcal toxic shock syndrome, thoracic surgery

## Abstract

**INTRODUCTION:**

Mediastinitis caused by group A *Streptococcus* (GAS) is extremely rare and often progresses rapidly, with high mortality.

**CASE PRESENTATION:**

A 43-year-old man with no significant medical history developed a sore throat. Several days later, he presented to a local clinic with a high-grade fever of 40°C and chest pain. He was initially advised to rest under observation, but his symptoms persisted. Two days later, he sought care at a nearby hospital, where chest CT revealed findings suggestive of mediastinitis. He was subsequently transferred to our hospital for emergency treatment. On arrival, he was in circulatory shock and showed signs of multiple organ dysfunction. Laboratory tests revealed markedly elevated inflammatory markers, including a C-reactive protein level of 40.0 mg/dL. Emergency surgical drainage of a mediastinal abscess was performed, followed by intensive care and antimicrobial therapy. Blood and pleural fluid cultures identified GAS as the causative organism. The patient’s condition gradually improved with continued systemic management. He was discharged from the ICU and began rehabilitation. Despite developing complications such as fingertip necrosis and delayed wound healing, he regained independent mobility. The patient was transferred to a rehabilitation facility on POD 48.

**CONCLUSIONS:**

This case highlights the importance of early recognition and immediate surgical and medical intervention in treating fulminant mediastinitis caused by GAS. Prompt and aggressive management can result in favorable outcomes, even in cases presenting with shock and multi-organ failure.

## INTRODUCTION

Mediastinitis is a life-threatening condition that typically occurs secondary to esophageal perforation, postoperative complications, or descending necrotizing infections.^1–3)^ Primary mediastinitis caused by group A *Streptococcus* (GAS) is extremely rare and is known to follow a fulminant course, often leading to streptococcal toxic shock syndrome (STSS).^4–6)^ In such cases, early diagnosis and aggressive therapeutic intervention are critical; however, owing to the rarity of this condition, optimal management strategies have yet to be established. Herein, we report a case of fulminant mediastinitis caused by GAS and complicated by STSS.

## CASE PRESENTATION

A 43-year-old man with no significant medical history developed a sore throat. Several days later, he presented to a local clinic with high-grade fever to 40°C and chest pain. He was initially advised to rest under observation, but his symptoms persisted. Two days later, he sought care at a nearby hospital, where chest CT revealed findings suggestive of mediastinitis. He was subsequently transferred to our hospital for emergency treatment.

**Fig. 1:**
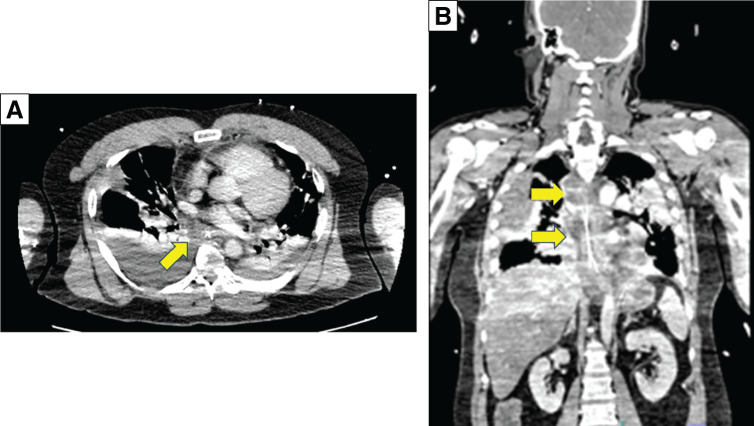
CT images of the chest demonstrating mediastinitis. (**A**) Axial contrast-enhanced chest CT shows increased fat density extending from the upper to the posterior mediastinum (yellow arrow). Bilateral pleural effusions are present, with loculated fluid collection on the right side. (**B**) Coronal contrast-enhanced chest CT confirming the extent of mediastinal inflammation and the presence of bilateral pleural effusions (yellow arrows).

On arrival, the patient presented in shock accompanied by severe respiratory distress, and marked purpura was noted at the fingertips. His vital signs included a body temperature of 39.1°C, blood pressure of 117/73 mmHg, and heart rate of 127 beats per minute. Laboratory tests revealed an elevated C-reactive protein level of 40.0 mg/dL and signs of renal and hepatic dysfunction, indicating multi-organ failure. Contrast-enhanced CT showed mediastinal widening and fat stranding along the esophagus without gas accumulation, along with bilateral pleural effusions, including loculated empyema on the right side (**Fig. 1**).

**Fig. 2:**
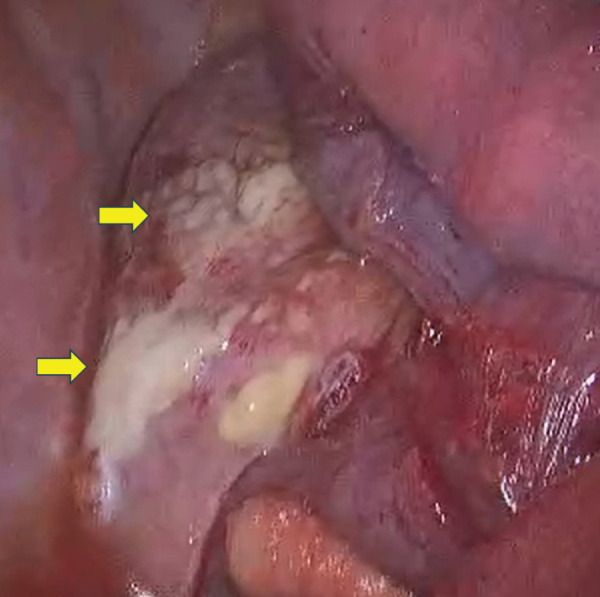
Intraoperative findings. Thoracotomy revealed extensive inflammation and fibrinous exudates (yellow arrows) on the mediastinal pleura of the right lower mediastinum.

Based on the CT findings, the infection was classified as type IIB, corresponding to a new classification of descending necrotizing mediastinitis (DNM), which is associated with a poor prognosis,^3)^ prompting us to proceed with emergency surgery. After confirming the absence of esophageal injury via upper gastrointestinal endoscopy, a thoracoscopic approach was initiated. However, owing to worsening ventilation parameters, the procedure was converted to a right posterolateral thoracotomy with sixth rib division, which allowed the surgery to be continued under bilateral lung ventilation. Extensive purulent fluid and inflammatory changes were observed in the mediastinum (**Fig. 2**), prompting mediastinotomy for adequate drainage. Once multiple drainage tubes were placed, surgery commenced. The operation lasted 2 hours and 26 minutes, with minimal blood loss. The patient was transferred to the ICU under mechanical ventilation.

In the ICU, the patient was treated with mechanical ventilation, broad-spectrum antibiotics, and hemodialysis (HD) for acute renal failure. The purpura of the fingertips progressively worsened, suggesting necrosis, and an acute-phase disseminated intravascular coagulation (DIC) score of 7 indicated that the fingertip necrosis was attributable to DIC. Antithrombin III concentrate and recombinant thrombomodulin therapy were initiated based on the acute-phase DIC score.

**Fig. 3:**
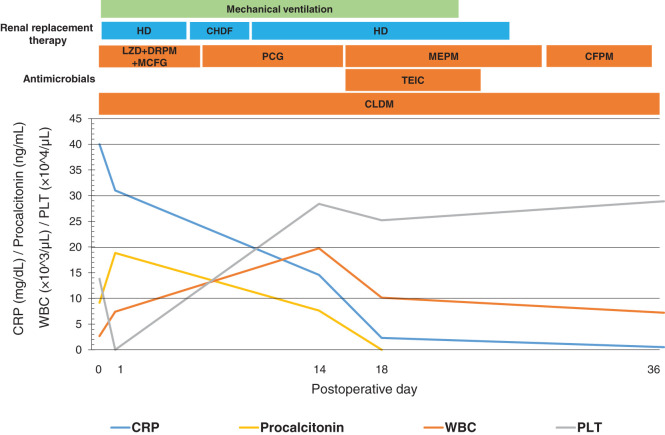
Post-hospitalization course. This graph illustrates the trends in inflammatory markers and major therapeutic interventions. Laboratory findings showed gradual improvement over time. CFPM, cefepime; CHDF, continuous hemodiafiltration; CLDM, clindamycin; CRP, C-reactive protein; DRPM, doripenem; HD, hemodialysis; LZD, linezolid; MCFG, micafungin; MEPM, meropenem; PCG, penicillin G; PLT, platelet count; TEIC, teicoplanin; WBC, white blood cell count

Antibiotic therapy with clindamycin, micafungin, doripenem, and linezolid was initiated on POD 1. On POD 4, blood and pleural fluid cultures grew GAS, and STSS was diagnosed. Antimicrobial susceptibility testing demonstrated sensitivity to penicillin G, meropenem, and clindamycin; therefore, micafungin, doripenem, and linezolid were discontinued, and the antimicrobial regimen was de-escalated to penicillin G. In addition, pleural fluid culture findings confirmed that the pleural effusion represented empyema secondary to mediastinitis rather than a reactive effusion; accordingly, pleural drainage was continued for an adequate duration to ensure sufficient source control. On the same day, because of worsening renal function and hemodynamic instability, renal replacement therapy was switched from intermittent HD to continuous hemodiafiltration. Subsequently, blood cultures obtained on POD 14 yielded *Klebsiella* species; therefore, teicoplanin was initiated, and penicillin G was replaced with meropenem. As the patient’s hemodynamic status stabilized, renal replacement therapy was converted back to intermittent HD on POD 20. After adequate drainage of the infectious focus was achieved and improvement in inflammatory markers was confirmed, the chest drains were removed on POD 23. Renal function further recovered, allowing discontinuation of HD on POD 24. CT demonstrated swelling of the right latissimus dorsi with increased attenuation of the surrounding fat and gas accumulation adjacent to the scapula, raising suspicion of necrotizing fasciitis; therefore, meropenem was replaced with cefepime on POD 26. All antimicrobial agents were discontinued after resolution of the infection was confirmed by laboratory findings, bacterial cultures, and CT on POD 46. By POD 48, the patient had regained independent ambulation and was transferred to a rehabilitation facility for further recovery (**Fig. 3**).

## DISCUSSION

This case report highlights a rare but critical manifestation of invasive GAS infection, resulting in mediastinitis and STSS. Although mediastinitis is typically associated with esophageal perforation, postoperative complications, or contiguous spread, hematogenous dissemination of GAS is an uncommon but important cause of severe infection, as previously reported in studies on DNM.^1–3)^ The rapid progression and high mortality rates of STSS (30%)^4,5)^ and DNM (ranging from 20% to 40%)^2,6)^ underscore the need for early recognition, timely intervention, and aggressive treatment strategies.

In the present case, the DNM was classified as type IIB based on the clinical course and chest CT findings, highlighting the importance of prompt and decisive surgical intervention.^3)^ Thoracoscopic exploration was initially attempted; however, owing to difficulty in maintaining adequate respiration under one-lung ventilation, conversion to open thoracotomy was required. Surgical interventions, including thoracoscopic exploration and thoracotomy, resulted in sufficient mediastinal debridement and drainage, thereby improving therapeutic outcomes. This case reaffirmed the critical role of surgical management in GAS-associated mediastinitis.^1,2)^

Antibiotic therapy was also a cornerstone of management in this case. Penicillin G was administered as the first-line agent, supplemented by meropenem and clindamycin to enhance bacterial eradication and inhibit exotoxin production, a critical factor in STSS pathophysiology.^7,8)^ Following the identification of GAS, empirical antimicrobial coverage was gradually de-escalated, illustrating the importance of initial broad-spectrum treatment followed by tailored therapy based on microbiological data.

In addition to the acute management approach, this case also demonstrated the need for continued care in the post-acute phase. Complications such as fingertip necrosis, delayed wound healing, and manifestations related to septic shock are well-documented sequelae of STSS. These complications, including DIC, necessitate multidisciplinary post-ICU care involving wound management, ongoing critical care, and rehabilitation.^9)^

In the present case, fingertip necrosis secondary to DIC was observed, underscoring the importance of a multidisciplinary approach encompassing wound management, intensive medical care, and rehabilitation.

Recent reports have noted an increasing incidence of invasive GAS infection, which may be related to changes in circulating GAS strains (e.g., the M1UK strain^10–12)^ after the COVID-19 pandemic) and host susceptibility, leading to severe disease even in individuals without apparent risk factors. Although we were unable to confirm that this case involved the M1UK strain, this possibility cannot be excluded given the clinical presentation and disease course.

We further emphasize that early radiologic assessment with multidetector CT is essential for establishing the diagnosis of DNM and delineating the extent of disease, thereby enabling timely and appropriate surgical planning and intervention.^13)^

## CONCLUSIONS

This case highlights the importance of early recognition and prompt surgical intervention in managing invasive GAS infections complicated by DNM and STSS. Timely, aggressive, and multidisciplinary treatment may improve survival in these life-threatening conditions.

## DECLARATIONS

### Availability of data and materials

Further details regarding the presented case are available from the corresponding author upon reasonable request.
